# Mechanisms of *Rostellularia procumbens* (L.) Nees on treating chronic glomerulonephritis explored by network pharmacology, RNA-seq, and in vitro experiments

**DOI:** 10.1186/s12906-023-04079-5

**Published:** 2023-07-24

**Authors:** Mengfan Wang, Yi Zhou, Qiuyuan Jian, Zhongzhu Ai, Shanshan Zhou

**Affiliations:** 1 School of Pharmacy, Hubei University of Chinese Medicine, Huangjiahu Road (West), Hongshan District, Wuhan, Hubei Province 430065 China; 2 Key Laboratory of Traditional Chinese Medicine Resources and Chemistry of Hubei Province, Hubei University of Chinese Medicine, Wuhan, 430065 China; 3 Modern Engineering Research Center of Traditional Chinese Medicine and Ethnic Medicine of Hubei Province, Hubei University of Chinese Medicine, Wuhan, 430065 China; 4 The First Clinical Medical School, Hubei University of Chinese Medicine, Huangjiahu Road (West), Hongshan District, Wuhan, Hubei Province 430065 China

**Keywords:** *Rostellularia procumbens* (L.) Nees, Chronic glomerulonephritis, Network pharmacology, RNA-seq, Mechanism

## Abstract

**Background:**

The purpose of this study was to demonstrate the in vitro anti-nephritis activity of *Rostellularia procumbens* (L.) Nees (*R. procumbens)* extract and to make a preliminary investigation of its anti-nephritis mechanism.

**Methods:**

A prediction network was built that describes the relationship between *R. procumbens* and CGN. Then, the potential targets for *R. procumbens* against CGN were imported into the DAVID database for Gene Ontology (GO) biological annotation analysis and Kyoto Encyclopedia of Genes and Genomes (KEGG) pathway analysis. A lipopolysaccharide (LPS)-stimulated rat mesangial cell HBZY-1 model in vitro was used to examine the anti-inflammatory activity of *R. procumbens* extract. RNA-seq was utilized to investigate differentially expressed genes (DEGs) and enriched signaling pathways between groups. Finally, qPCR was used for the validation analysis of the experimental results.

**Results:**

The results of network pharmacology showed that *R. procumbens* exerts its therapeutic effect on CGN through the AGE-RAGE signaling pathway in diabetic complications, PI3K-Akt, IL-17 signaling pathway, and so on. *R. procumbens* n-butanol extract (J-NE) can effectively relieve inflammation in HBZY-1. The results of KEGG pathway enrichment suggest that J-NE attenuated CGN was associated with the IL-17 signaling pathway, and the results of RNA-seq were consistent with network pharmacology. Targets enriched in the IL-17 signaling pathway, including Chemokine (C-C motif) ligand 7 (CCL7), Lipocalin 2 (LCN2), Chemokine (C-C motif) ligand 2 (CCL2), and Chemokine (C-X-C motif) ligand 1 (CXCL1), have been identified as crucial targets attenuating CGN by J-NE.

**Conclusion:**

*R. procumbens* is a promising pharmacological candidate for the treatment of CGN in the present era.

**Supplementary Information:**

The online version contains supplementary material available at 10.1186/s12906-023-04079-5.

## Background

Chronic kidney disease is defined as decreased glomerular filtration rate, increased urinary albumin excretion, or both, and is a growing public health problem. The global prevalence is estimated at 8–16% [1]. CGN accounts for about 20% of chronic kidney disease cases in most countries, and in young adults, glomerulonephritides are the most frequent cause of end-stage renal disease [2]. A patient with end-stage renal failure must receive dialysis or kidney transplantation to survive for more than a few weeks (Johns Hopkins Medicine, 2020, https://www.hopkinsmedicine.org/health/conditions-and-diseases/end-st-age-renal-failure, accessed 18 May 2022) [3], which could significantly affect the quality of life and greatly increase the economic burden for patients. The development of kidney biopsy makes possible such as histopathology and ultrastructural observation. CGN can be divided into minimal change disease, membranous nephropathy, mesangial proliferative glomerulonephritis, membranoproliferative glomerulonephritis, and focal segmental glomerulosclerosis according to the pathological examination results. Medical treatment is mainly based on glucocorticoids and immunosuppressive agents, including prednisone, cyclophosphamide (CTX), cyclosporine A, mycophenolate mofetil, leflunomide, rituximab, and so on [4]. To address causes, diuretics, antihypertensive therapy, anticoagulation therapy, and treatment of blood lipids were implemented as well. However, the clinical use of these drugs had large adverse reactions and side effects, which are easy to develop resistance to. Doctors should strictly grasp the indications, usage, and dosage and carefully weigh the efficacy and treatment risk when using them, which have high requirements for doctors and patients.

Therefore, we should actively seek treatment for CGN to prevent the disease from continuing to a stage that requires dialysis or kidney transplantation.

Information about *R. procumbens*, a medicinal plant of the Acanthaceae family, was first published in Sheng Nong’s Herbal Classic. The plant name has been checked with The Plant List (http://www.theplantlist.org, accessed 14 June 2022). It is mainly composed of lignans, flavones, and triterpenes [5], which are clinically used to treat colds, fevers, flu, malaria, and hepatitis. Studies have demonstrated that multiple bioactive effects, including antitumor [6–8], anti-chronic glomerulonephritis [9], antiplatelet aggregation [5, 10, 11], and antiviral [12, 13] activities are exerted by active compositions of *R. procumbens* [14].

In this study, network pharmacology was used to predict the potential pharmacodynamic components and effective mechanism of J-NE in the treatment of CGN. RNA-seq was used to comprehensively analyze the mechanism of action of J-NE in the treatment of CGN, and qPCR was used for final verification.

## Materials and methods

### Compounds collection and screening

The main compounds contained in *R. procumbens* were retrieved from relevant China and foreign literature, and a database of *R. procumbens* compounds was established [15]. Pharmacokinetics methods were used to screen compounds [16].

### Targets collection

The SMILES molecular formulas of candidate active compounds were obtained from the PubChem database (https://pubchem.ncbi.nlm.nih.gov/) and imported into the SwissTargetPrediction database (http://www.swisstargetprediction.ch/) to predict potential target information. The predicted targets were ranked according to their scores, and a threshold of 10 was chosen to screen approximately 18% of the targets as potential targets for candidate compounds.

The GeneCards database (https://www.genecards.org/) was searched using the keyword “chronic glomerulonephritis” to obtain targets associated with CGN.

### Construction of a protein-protein interaction (PPI) network

The predicted targets of candidate compounds were compared and crossed with the collected “chronic glomerulonephritis” targets to obtain the potential targets of *R. procumbens* against CGN and to construct a PPI network of potential targets of *R. procumbens* for the treatment of CGN. To construct PPI networks, these possible targets were loaded into the STRING database (https://string-db.org/). The networks were then loaded into the Cytoscape 3.7.2 software, which was used to examine the close relationships between the targets.

### Enrichment analysis

The targets were entered into the DAVID database (https://david.ncifcrf.gov/summary.jsp) for GO functional annotations and KEGG pathways enrichment analysis. Biological process, molecular function, and cellular component were selected for GO functional annotations. *p* ≤ 0.05 was set as the threshold to screen for significantly different terms or pathways.

### Network construction

According to the predicted anti-CGN potential targets and the results of enrichment analysis of KEGG, the Cytoscape 3.7.2 software was used to construct a network diagram of the Drug-Compounds-Targets-Disease.

### Materials and reagents

*Rostellularia procumbens* (L) Nees. was harvested in October 2019 from the Wuchang district, Hubei province of China, and was authenticated by Prof. Hezhen Wu, Hubei University of Chinese Medicine (HBUCM). Collection of plant materials complied with local or national guidelines. Voucher specimens (JC-2019-01) were stored in the pharmaceutical college.

Petroleum ether, ethyl acetate, n-butanol, and DMSO (Analytical Reagent) were purchased from Sinopharm Chemical Reagent Co., Ltd (Shanghai, China). The ethanol used for extraction was food grade. LPS (L4391) was purchased from Sigma (USA). The Rat TNF-α Elisa Kit (E-EL-R2856c) was purchased from Elabscience Biotechnology Co., Ltd (Wuhan, China). MTT (1334) was purchased from Bioforxx (Germany).

### Obtaining *R. procumbens* extract

Fifteen kg of *R. procumbens* (obtained from Key Laboratory for Traditional Chinese Medicine Resources and Chemistry of Hubei Province) was weighed, crushed, sieved (24 mesh), and percolated with ten volumes of 80% ethanol to obtain a percolation liquid. The resulting diafiltrate was concentrated to remove ethanol under reduced pressure. Through systematic solvent extraction, the *R. procumbens* is finally divided into petroleum ether extraction material (J-PE), ethyl acetate extraction material (J-EE), and water-saturated n-butanol extraction material (J-NE).

### Cell culture

Rat kidney mesangial cells (HBZY-1) were supplied by China Center for Type Culture Collection (CCTCC, Wuhan, China), and were cultured in a humidified incubator at 37℃ containing 5% carbon dioxide using DMEM (Thermofisher, USA) supplemented containing 100 U/mL penicillin/streptomycin mixture (BasalMedia, Shanghai, China) and 10% fetal bovine serum (Thermofisher, USA). When growth to 80–90% confluence, cells were washed once with phosphate-buffered saline (PBS) and digested by 0.25% trypsin (with 0.38 g/L EDTA, BasalMedia, Shanghai, China), spare. Every fraction from *R. procumbens* was dissolved in DMSO (MP Biomedicals, USA) to prepare a 200 mg/mL stock solution. It was diluted to the target concentration with DMEM when used (The concentration of DMSO in each group is less than 0.5%).

### Cell proliferation assay

Cell proliferation was detected by 3-(4,5-dimethylthiazole-2yl)-2,5-diphenyltetrazolium bromide (*MTT*) assay. HBZY-1 cells were seeded in 100 µl media at 4000 cells/well in 96-well plates with three parallel wells in each group. After the cells adhered, fractions were added to each well at a final concentration of 50, 100, 200, and 400 µg/mL and then treated for 24 h. After cells were incubated with MTT solution (0.5 mg/mL) for 4 h at 37 °C, discarding the cell supernatant containing MTT, each well was added 150 µl DMSO. The 96-well plate was shaken with a plate shaker to dissolve the formazan, and the optical density (OD) was measured at the absorption wavelength of 490 nm.

### Screening of concentration of LPS acting on HBZY-1 cells

HBZY-1 cells were seeded into a 6-well plate at 3 × 10^5^ cells/well, and LPS with a concentration of 0.5, 1, 2, 3, 4, and 5 µg/mL was added after adhering to the wall. After 24 h of treatment, the Elisa kit was used to detect the TNF-α concentration of the supernatant.

### Anti-inflammatory effect of each fractions

HBZY-1 cells were seeded into 6-well plates at 3 × 10^5^ cells/well. The control, model and experimental group (J-PE, J-EE, and J-NE) were set up. After the cells adhered, the model and the experimental group were induced with 1 µg/mL LPS for 24 h. Then the experimental group was treated with each fraction (50, 100 µg/mL) for 24 h. The supernatant was taken for TNF-α concentration measurement by Elisa kit.

### Transcriptomic analysis

HBZY-1 cells were seeded into 6-well plates at 3*10^5^ cells/well. The control, model, and experimental groups (J-NE, 100 µg/mL) were set up. After modeling and administration in the same way as in *2.11*, the supernatant was discarded, 1 mL of TRIzol reagent (Biosharp, China) was added to each well to digest and lyse the cells, and the cells were collected with an enzyme-free EP tube.

Total RNA was extracted according to the requirements of the instructions, and the qualified total RNA test by Agilent Bioanalyzer 2100 (Agilent Technologies, USA) was the starting sample for mRNA sequencing. The starting total RNA was accurately quantified with QUBIT RNA ASSAY KIT (Invitrogen, USA). The purified and fragmented total RNA was used to build a cDNA library, and the quality of the cDNA library was checked using an Agilent Bioanalyzer 2100. Q-PCR method was used to accurately quantify the effective library concentration (library effective concentration > 2 nM). Qualified samples were sent to the Illumina Hiseq2500 platform for sequencing to obtain sequencing data.

The reference genome version of this project was Rnor_6.0. After filtering the original sequencing data, the alignment software Star^2^ 2.7.0d (https://github.com/alexdobin/STAR) was used to map clean reads to the reference genome. According to the bam files obtained by the alignment, QoRTs^3^ 1.3.0 (http://hartleys.github.io/QoRTs/) was used to perform statistics and graphs. The quality of the sequencing data was evaluated from the aspects of alignment rate, alignment region, and gene region coverage.

DESeq^5^ was used to analyze and screen out DEGs (screening conditions: |log2Fold Change | >1, qvalue < 0.05). We use bioinformatics (http://www.bioinformatics.com.cn/) to draw volcano plots and Venn diagrams of DEGs. The Venn diagram was used to count the number of DEGs in common between each group. DEGs in common were uploaded to the DAVID database to analyze GO terms and KEGG pathways. Then identify the signaling pathways that DEGs are mainly involved in.

### qPCR

Total RNA was reverse transcribed to cDNA using the RevertAid First Strand cDNA Synthesis Kit (Thermofisher, USA). qPCR was performed using TB Green® Premix Ex Taq™ II (TAKARA, Japan). Samples were analyzed in triplicate and normalized by subtracting the average Ct value of the sample from the average Ct value of the housekeeping gene GAPDH. Primer information is shown in the supplementary file (Table S3).

### Statistical analysis

GraphPad Prism 8.0 (GraphPad Software Inc., California, USA) was used for statistical analysis. Statistical differences between groups were evaluated by one-way analysis of variance (ANOVA). *p* < 0.05 was considered statistically significant.

## Result

### Network pharmacology

In this research, twenty-eight compounds were screened out and used as candidate active compounds. Twenty-eight candidate active compounds were shown in the supplementary file (Table S1).

**Fig. 1:**
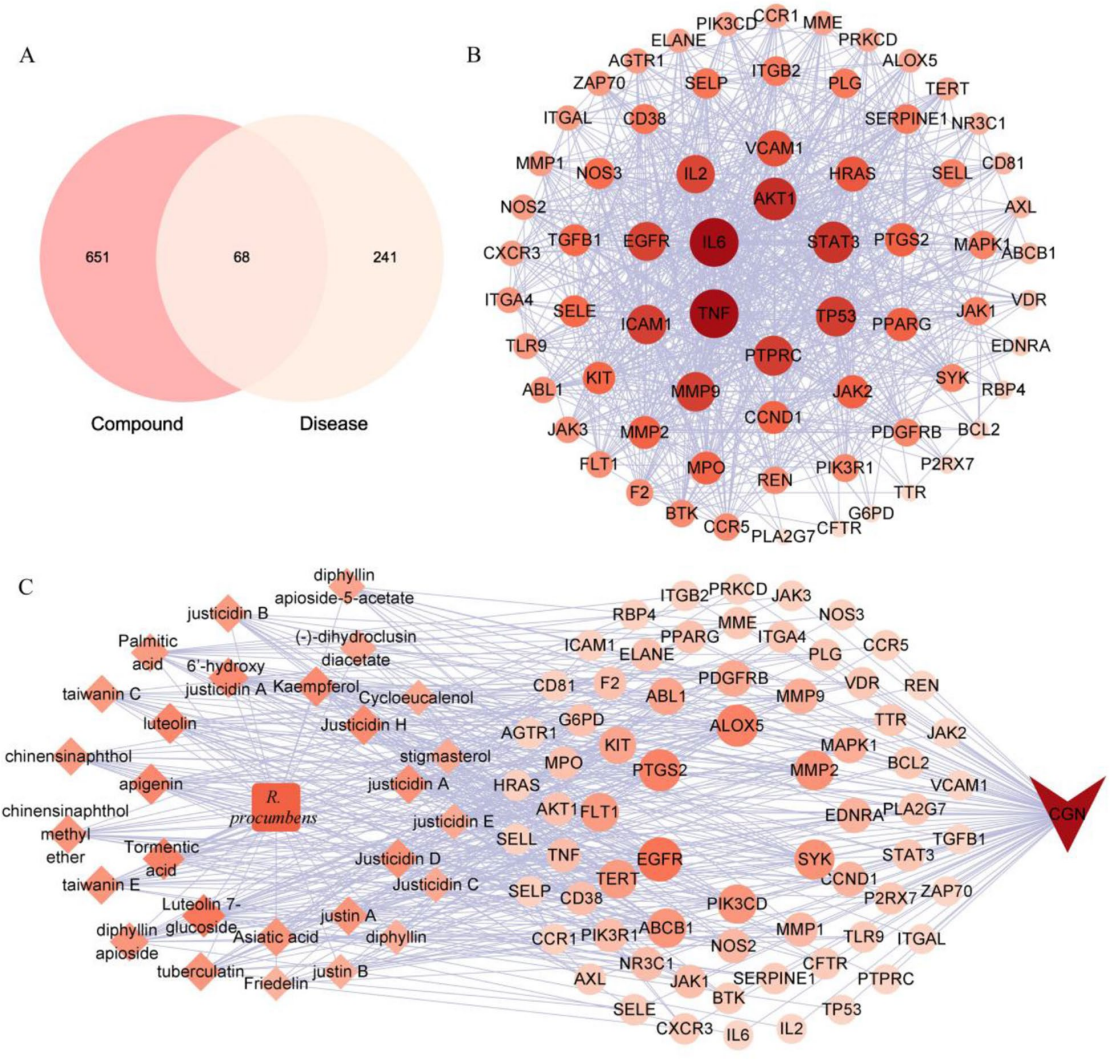
Network pharmacology study of *R. procumbens* attenuating CGN. **A** Venn diagram. The intersection of the predicted targets of candidate active compounds in *R. procumbens* and CGN-related disease targets. **B** PPI network of the common target. The colors of the nodes are illustrated from dark red to orange to flesh color in descending order of degree values. Nodes size are illustrated in descending order of degree values. **C** Drug-Compounds-Targets-Disease network of *R. procumbens* attenuating CGN. The square node represents Drug. The diamond nodes represent Compounds. The circle nodes represent Targets. The arrow node represents Disease. The colors of the nodes are illustrated from dark red to orange to flesh color in descending order of degree values. Nodes size are illustrated in descending order of degree values

Three hundred and nine targets related to CGN were obtained and compared with the predicted targets of candidate active compounds. Sixty-eight potential targets (Table S2) for *R. procumbens* against CGN were obtained (Fig. 1A). The names of these 68 targets and their interaction relationships were displayed in the PPI network (Fig. 1B). It could be seen that TNF, IL6, AKT1, STAT3, TP53, and PTRPC occupy key positions in the network. It suggested that they may be key targets for drug intervention.

The Drug-Compounds-Targets-Disease network comprised 98 nodes and 470 edges and demonstrated the relationship of the compounds of *R. procumbens* to diseases and shared targets (Fig. 1C). Figure 1C showed that TNF was intervened by tormentic acid, luteolin 7-glucoside, justin A, justin B, dihydroclusin diacetate, and cycloeucalenol. IL6 was intervened by asiatic acid. AKT1 was intervened by apigenin, luteolin, taiwanin c, kaempferol, and justicidin E. STAT3 was intervened by 6′-hydroxy justicidin A, justicidin H, taiwanin E, and friedelin. TP53 was intervened by luteolin 7-glucoside. PTRPC was intervened by palmitic acid.

**Fig. 2:**
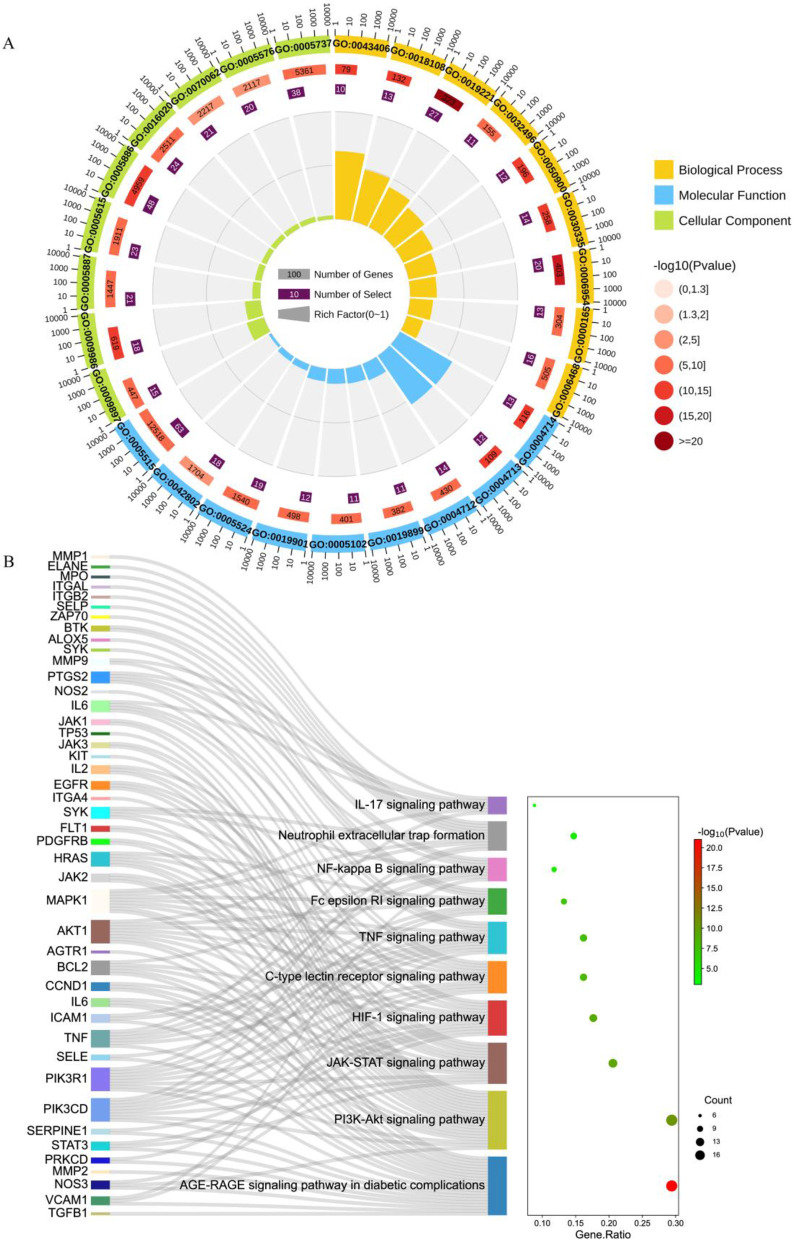
**A** GO annotation analysis of *R. procumbens* attenuating CGN. The outermost circle: the classification. Outside the circle is the scale of the number of genes, and different colors represent different classifications. The second circle: the number and the *P*-value of the class in the background gene. The more genes, the longer the bar, the smaller the *P*-value, and the redder the color. The third circle: the total number of differential genes. The fourth circle: the RichFactor value of each class (the ratio of differential genes in the class to the total number of genes in the class), and each cell of the background auxiliary line represents 0.1. **B** KEGG pathway enrichment analysis of *R. procumbens* attenuating CGN. The left column is the gene name, and the connecting line indicates the affiliation

Figure 2A depicted the enriched biological processes of the targets, which were mainly associated with a cytokine-mediated signaling pathway, inflammatory responses, and protein phosphorylation. The cellular components were mainly distributed in the plasma membrane, cytoplasm, and membrane. As to the enriched molecular functions, the target proteins were mainly connected with protein binding, ATP binding, and identical protein binding. Figure 2B showed a KEGG pathway enrichment analysis of 68 potential functional targets. The results indicated that *R. procumbens* exerted protective effects against CGN and was closely related to the ten pathways, including the PI3K-Akt signaling pathway, NF-kappa B signaling pathway, and IL-17 signaling pathway.

### Cell proliferation assay

**Fig. 3:**
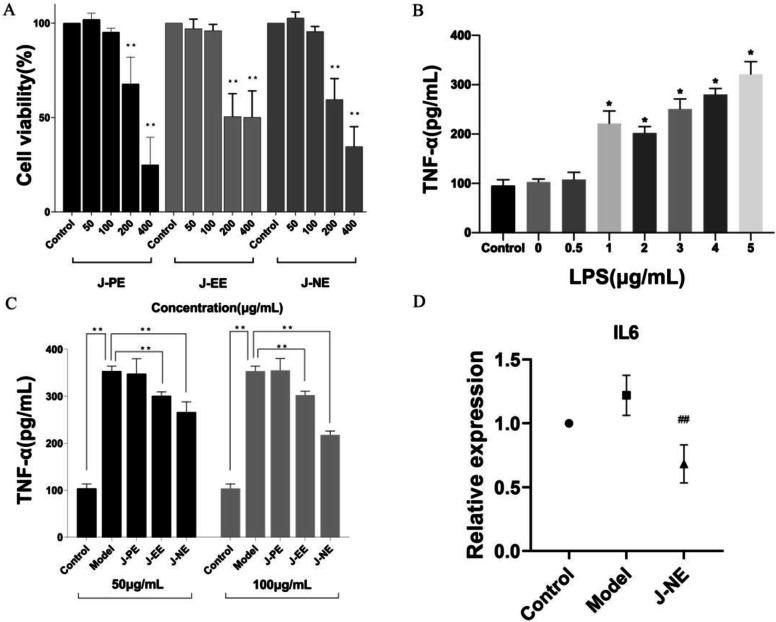
Pharmacodynamics study of *R. procumbens* attenuating CGN. Data represents mean ± standard deviation. **A** Effect of different fractions on HBZY-1 cells viability, ^**^p < 0.01. **B** Different concentrations of LPS induced HBZY-1 cells to produce TNF-α, ^*^p < 0.05. **C** Different concentrations of different fractions acted on LPS-induced HBZY-1 cells to produce TNF-α, ^**^p < 0.01. **D** The relative expression of IL6 in each group. Significantly different compared to the control group: ^**^p < 0.01

The safe dose of J-PE, J-EE, and J-NE was first determined, as shown in Fig. 3A. After different concentrations of J-PE, J-EE, and J-NE treated on HBZY-1 for 24 h, compared with the control group, there was no significant difference in cell viability when these fractions were less than 100 µg/mL. Therefore, 50 µg/mL and 100 µg/mL were selected for follow-up studies.

### Screening of concentration of LPS acting on HBZY-1 cells

To investigate the appropriate concentration of LPS, we assayed the conditioned medium using a TNF-α ELISA kit. When HBZY-1 was incubated with 0.5, 1, 2, 3, 4, and 5 µg/mL of LPS, compared with the control group, the secretion of TNF-α was significantly different (*p* < 0.05) (Fig. 3B). It showed that a large number of inflammatory factors were produced after the intervention of LPS. TNF-α levels increased with increasing concentrations of LPS, so the follow-up experiments were carried out under the condition that LPS concentration was 1 µg/mL and the action time was 24 h.

### Anti-inflammatory effect of fractions of *R. procumbens*

Figure 3C showed that compared with the control group, the concentration of TNF-α in the model group was significantly increased (*p* < 0.01), while the TNF-α concentration after 100 µg/mL J-NE treatment was significantly decreased (*p* < 0.01). The J-EE also had the effect of reducing the secretion of TNF-α, but its effect is not as good as that of J-NE. Therefore, we considered that the active ingredients for the treatment of CGN are concentrated in the n-butanol fraction (J-NE).

### Transcriptomic analysis

**Fig. 4:**
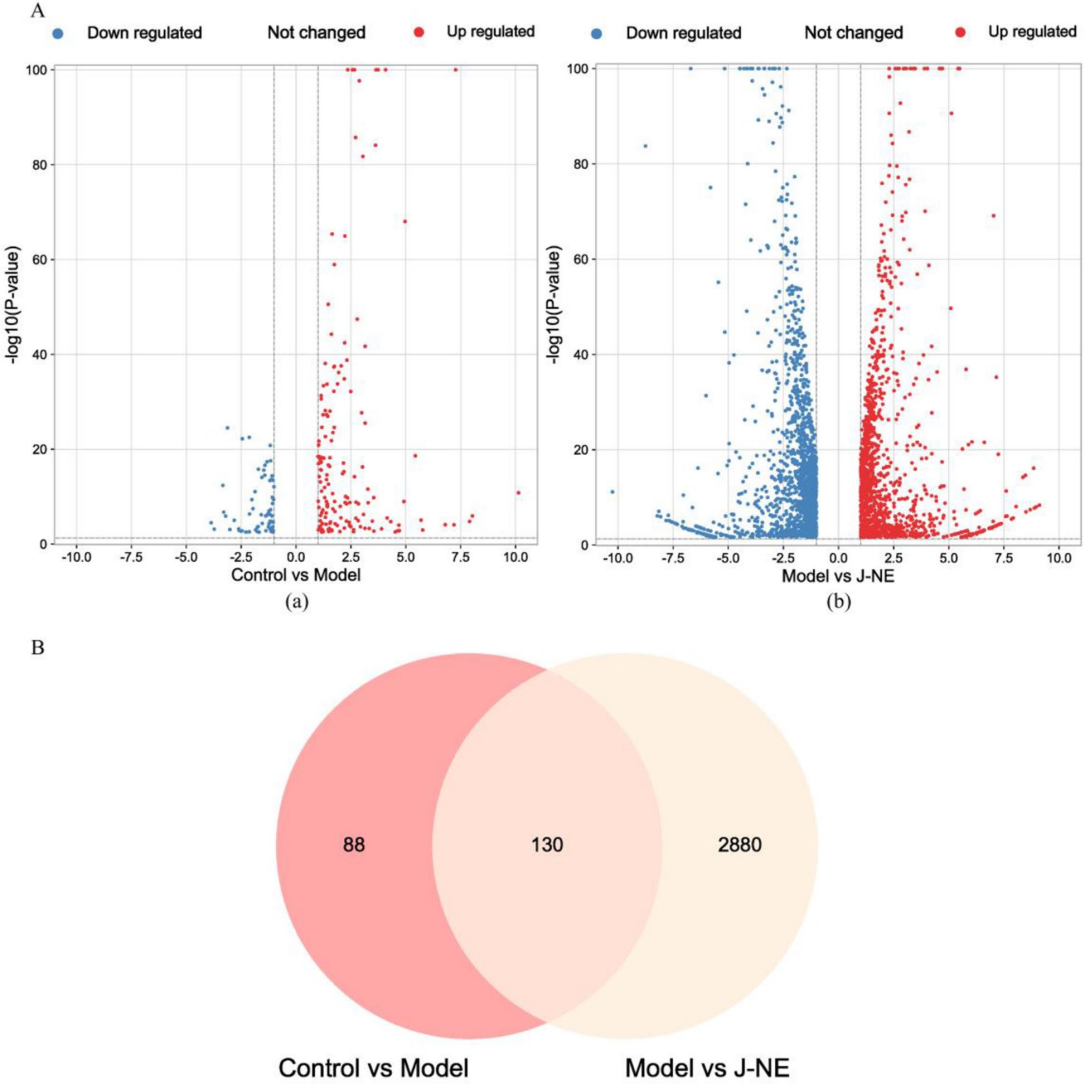
Transcriptome study of *R. procumbens* attenuating CGN. A The volcano map. (a) Control vs. Model, (b) Model vs. J-NE. B Venn diagram. The intersection is genes with expression changes induced by J-NE treatment

As shown in the volcano plot (Fig. 4A), compared with the control group, 218 DEGs were screened from the model group, of which 153 were up-regulated, and 65 were down-regulated. Compared with the model group, 3010 DEGs were screened from the experimental group, of which 1374 were up-regulated, and 1636 were down-regulated.

**Fig. 5:**
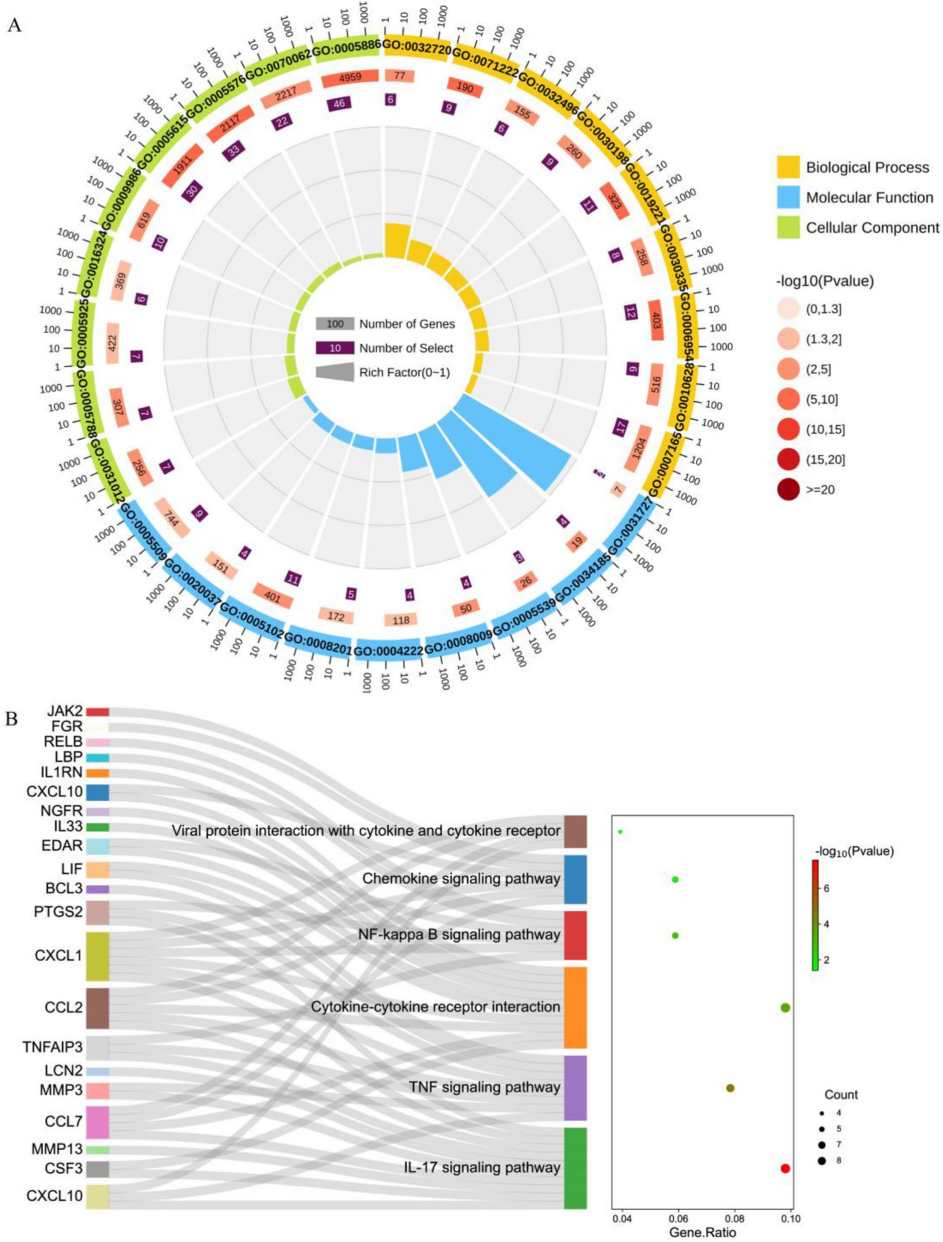
**A** KEGG pathway enrichment analysis of *R. procumbens* attenuating CGN. The left column is the gene name, and the connecting line indicates the affiliation. **B** GO annotation analysis of *R. procumbens* attenuating CGN. The outermost circle: the classification. Outside the circle is the scale of the number of genes, and different colors represent different classifications. The second circle: the number and the *P*-value of the class in the background gene. The more genes, the longer the bar, the smaller the *P*-value, and the redder the color. The third circle: the total number of differential genes. The fourth circle: the RichFactor value of each class (the ratio of differential genes in the class to the total number of genes in the class), and each cell of the background auxiliary line represents 0.1

**Fig. 6:**
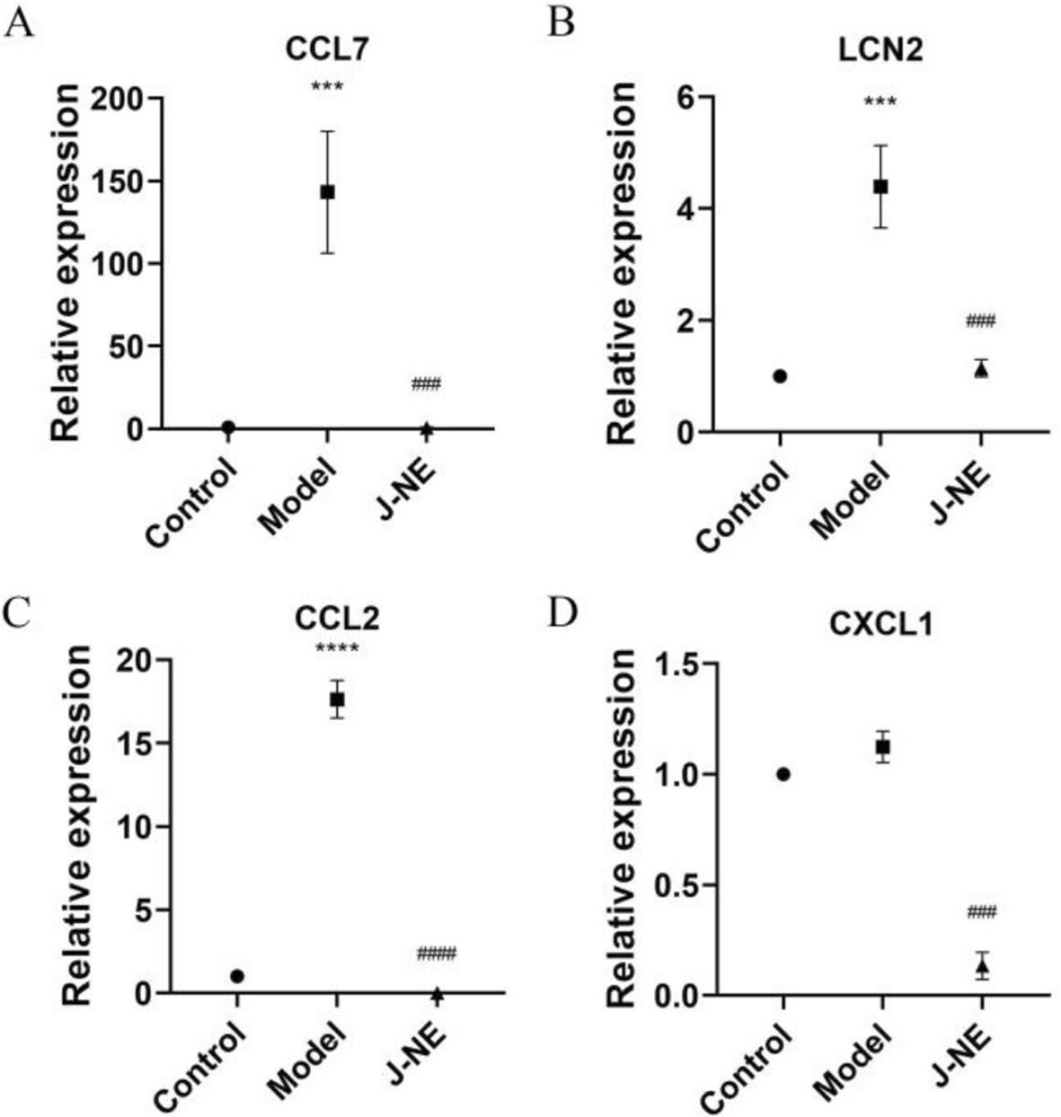
qPCR verification. **A**, **B**, **C** and **D** show relative expression of CCL7, LCN2, CCL2 and CXCL1, respectively. Data represents mean ± standard deviation. Significantly different compared to the control group: ^***^p < 0.001, ^****^p < 0.0001. Significantly different compared to the model group: ^###^*p* < 0.001, ^####^*p* < 0.0001

Figure 4B showed the 130 genes with expression changes induced by J-NE treatment, which may be a key target of J-NE in CGN remission. Figure 5A depicted the enriched biological processes of the targets, which were mainly associated with signal transduction, inflammatory responses, and cytokine-mediated signaling pathways, two of which were consistent with the network pharmacology results. The cellular components were mainly distributed in the plasma membrane, extracellular region, and extracellular space, one of which was consistent with the network pharmacology results. As to the enriched molecular functions, the target proteins were mainly connected with receptor binding, calcium ion binding, and heparin binding. The results of KEGG pathway enrichment (Fig. 5B) indicated that *R. procumbens* exerted its effects in the relief of CGN and was closely related to the six pathways, including the IL-17 signaling pathway, Cytokine-cytokine receptor interaction, and NF-kappa B signaling pathway. The IL-17 signaling pathway was the one we were interested in and ranked first according to the *p*-value, which was also consistent with the results of network pharmacology. Therefore, we considered that the targets enriched in the IL-17 signaling pathway including CXCL10, CSF3, MMP13, CCL7, MMP3, LCN2, TNFAIP3, CCL2, CXCL1, and PTGS2 were targets for J-NE against CGN. We selected some genes and verified their expression at the transcriptional level using qPCR (Fig. 6). The changes in the transcript levels of CCL7, LCN2, and CCL2 were consistent with the tendencies observed in the RNA-seq analysis. The changes of CXCL1 at the transcriptional level were consistent with some literature studies.

## Discussion

Traditional Chinese medicine and pharmacology are a great treasure-house of the Chinese nation and a glorious crystallization of its great culture. Just as ancient TCM books offer clues for Ms. Tu Youyou to discover artemisinin, we have discovered a TCM that can relieve nephritis, which name is *Rostellularia procumbens* (L.) Nees.

By employing the network pharmacology method, we preliminarily predicted the mechanism of *R. procumbens* attenuating CGN. Through KEGG enrichment analysis, it was concluded that *R. procumbens* might act through the PI3K-Akt, NF-kappa B, IL-17 signaling pathway, and so on. However, it is necessary to further clarify in experiment ways whether *R. procumbens* affects as ancient TCM books say and whether it works through pathways analyzed in network pharmacology. In this study, based on network pharmacology, further analysis was performed using RNA-seq, and we found J-NE may have a therapeutic effect on CGN via influencing the expression of CCL7, LCN2, CCL2, and CXCL1, consequently changing the inflammatory response and controlling the IL-17 signaling pathway. qPCR was used for the validation analysis of the experimental results.

CGN is a kind of disease that includes many pathological types, is caused by a variety of factors, and originates in the glomerulus. A part of chronic nephritis occurs because of the continuation of acute nephritis, but most of them are caused by immune complexes deposition in the glomerulus. These immune complexes directly promote glomerular inflammation and mesangial hyperplasia. Activation of the local and systemic renin-angiotensin system and complement also leads to glomerulosclerosis and tubulointerstitial fibrosis, which leads to loss of renal function [17]. Mesangial cells within the glomerulus help regulate glomerular filtration, phagocytosis of immune complexes, and extracellular matrix production [18]. Thus, the rat glomerular mesangial cell line, HBZY-1, was used for our studies. Mesangial cells produce cytokines, and chemokines when activated by immune or inflammatory stimuli [19]. Several studies have found that LPS or other factors, such as polymeric immunoglobulin A1 (pIgA1) and hyperglycemia, can activate mesangial cells to produce pro-inflammatory cytokines, including IL-6, transforming growth factor-beta 1 (TGF-beta 1), TNF-α and NO [20–22]. Our study selected readily available LPS as the inducer. Therefore, in this study, the rat mesangial cell HBZY-1 model stimulated by lipopolysaccharide was used to study the effect of J-NE on CGN in vitro.

In addition to measuring the concentration of TNF-α in the cell supernatant, we also examined the expression of IL-6 at the transcriptional level. As seen in Fig. 3C and D, TNF-α was significantly up-regulated in LPS-stimulated HBZY-1 cells as well as IL-6, indicating that the in vitro CGN model was successfully established.

Three different polarities of *R. procumbens* extract were used to treat LPS-stimulated HBZY-1 cells. We found that J-NE was significantly effective in the in vitro treatment of CGN. Three cell groups including control group, model group, and J-NE (100 µg/mL) group were set up, and the possible mechanism of J-NE against CGN using RNA-seq technology was explored. The IL-17 signaling pathway was the one we were interested in and ranked first according to the *p*-value, which was also consistent with the results of network pharmacology. The targets enriched in the IL-17 signaling pathway, including CCL7, LCN2, CCL2, and CXCL1, were targets for J-NE against CGN.

Mesangial cells were involved in glomerular inflammation because they are equipped with a set of innate pathogen-recognition receptors that enable them to recognize a large number of pathogen-associated molecular patterns and damage-associated molecular patterns and translate the recognition of these danger signals into the pro-inflammatory secretion of mediators, namely cytokines and chemokines [23], such as MCP-1/CCL2 [24–26], MCP-3/CCL7 [26], and CXCL1 [24, 27], which promoted the recruitment of macrophages to the mesangium, thereby accelerating inflammation and damage in glomerular [27]. Naotoshi Kanemitsu et al. showed that the glutaminyl cyclase (QC)/isoQC inhibitor PQ529 could inhibit the CCL2/CCR2 axis to suppress the progression of renal dysfunction in rats with glomerulonephritis [25]. A clinical study showed that urinary MCP-1 excretion was higher in patients with chronic glomerulonephritis than in healthy controls. There was a correlation between urinary MCP-1 and the severity of tubulointerstitial fibrosis [28]. In our study, we experimentally confirmed that J-NE, an extract from *R. procumbens*, could reduce the mRNA expression level of MCP-1/CCL2, MCP-3/CCL7, and CXCL1 induced by LPS, which inhibits inflammation.

LCN2, also known as neutrophil gelatinase-associated lipocalin (NGAL), was mainly expressed in neutrophils and played an important role in innate immunity [29]. It was also expressed in hepatocytes, renal tubular cells [29], and mesangial cells [30, 31]. Studies have shown that LCN2 promotes Th1 cell differentiation in an autocrine or paracrine manner through the IL-12/STAT4 pathway, leading to lupus nephritis (LN) deterioration. During the pathological exacerbation of LN, the kidney (tubular epithelial cells and infiltrating leukocytes) can also act as a source of LCN2, forming a positive feedback loop [32]. Atsushi Hashikata et al. identified and confirmed up-regulated genes and their products in mesangial cells co-cultured with endotoxin-stimulated macrophages and found that inflammatory stimulation led to increased LCN2 production in mesangial cells without significant cellular damage. Thus, they speculated that LCN2 levels may be related to renal dysfunction rather than the damage itself. Their in vitro experiments also showed that LPS and TNF-α synergistically up-regulated the expression of the LCN2 gene. Thus, infiltrating macrophage-derived TNF-α and toll-like receptor-4 ligand synergistically up-regulated mesangial LCN2 gene expression [30]. The increase of bone-production of fibroblast growth factor 23 (FGF23) contributed to cardiovascular death in CKD. Renal production of LCN2 mediates inflammation and increased bone production of FGF23 in CKD. Guillaume Courbon et al. found that inhibition of LCN2 may be a potential therapeutic approach to reduce FGF23 and improve CKD outcomes [33]. In our study, we experimentally confirmed that J-NE could reduce the secretion of TNF-α and the mRNA expression level of LCN2 induced by LPS, which inhibits inflammation.

Here, we demonstrated that J-NE inhibits the production of TNF-α and the expression level of MCP-1/CCL2, MCP-3/CCL7, and CXCL1, thus providing a potential mechanism for its anti-inflammatory activity. A previous study in our laboratory found that treatment with *R. procumbens* extracts significantly ameliorated ADR-induced renal pathological changes in mice and that the therapeutic mechanism of J-NE was associated with the inhibition of podocyte apoptosis [9]. This study explored the inflammatory model, which complements the multicellular study of J-NE on chronic glomerulonephritis. It also provides a potential therapeutic agent for the treatment of chronic glomerulonephritis.

Regrettably, we detected changes in chemokine expression levels in a single cell model in vitro that elicit responses from other populations of cells if in vivo. Although our findings may not be the direct cause of the reduction in inflammatory factors in vitro, they were undoubtedly surprising findings pointing to future research directions.

## Conclusion

J-NE can have a therapeutic effect on CGN by affecting the expression of CCL7, LCN2, CCL2, and CXCL1, thereby altering the inflammatory response and controlling the IL-17 signaling pathway, and is a promising drug candidate for the treatment of CGN.

## Supplementary Information

**Additional file 1: Table S1.** 28 candidate active compounds for Network pharmacology. (Note: Pharmacokinetic data below are referenced from the article by Xie et al. [15]).** Table S2. **68 potential targets for *Rostellularia procumbens* (L.) Nees against chronic glomerulonephriti. **Table S3. **Primer information.

## Data Availability

All data generated or analysed during this study are included in this published article [and its supplementary information files].
