# Supplementary material for: Mechanisms of Rostellularia procumbens (L.) Nees on treating chronic glomerulonephritis explored by network pharmacology, RNA-seq, and in vitro experiments
Source: BMC Complement Med Ther. 2023 Jul 24;23:263. doi: 10.1186/s12906-023-04079-5 (PMC10367255; doi:10.1186/s12906-023-04079-5)
Supplement: Supplementary file 1 — Additional file 1: Table S1. 28 candidate active compounds for Network pharmacology. (Note: Pharmacokinetic data below are referenced from the article by Xie et al. [15]). Table S2. 68 potential targets for Rostellularia procumbens (L.) Nees against chronic glomerulonephriti. Table S3. Primer information. [file 12906_2023_4079_MOESM1_ESM.docx]

**Table S1**

| **Components** | **HIA** | **Caco-2** | **PPB** |
| --- | --- | --- | --- |
| Stigmasterol | 100 | 52.34 | 100 |
| Cycloeucalenol | 100 | 50.46 | 100 |
| Friedelin | 100 | 46.48 | 100 |
| Palmitic acid | 98.3 | 26.07 | 100 |
| Luteolin | 79.43 | 4.54 | 99.72 |
| Apigenin | 88.12 | 10.55 | 97.25 |
| Asiatic acid | 91.24 | 20.98 | 96.46 |
| Tormentic acid | 91.23 | 20.9 | 96.03 |
| Justicidin E | 97.75 | 29.66 | 89.95 |
| Kaempferol | 79.44 | 9.58 | 89.61 |
| Justicidin D | 98.3 | 33.12 | 89.42 |
| Taiwanin C | 97.75 | 29.66 | 89.42 |
| Justicidin C | 98.14 | 42.34 | 89.19 |
| Justicidin B | 97.64 | 38.68 | 88.97 |
| Justicidin A | 98.14 | 42.34 | 88.74 |
| Justin A | 97.2 | 28.91 | 88.38 |
| Taiwanin E | 96.81 | 21.87 | 88.29 |
| Chinensinaphthol | 96.74 | 24.35 | 88.1 |
| Chinensinaphthol methyl ether | 96.74 | 24.35 | 88.1 |
| Diphyllin | 96.74 | 24.3 | 88.07 |
| Justicidin H | 96.74 | 24.3 | 87.59 |
| Dihydroclusin diacetate | 99.19 | 48.85 | 87.47 |
| Justin B | 96.83 | 32.24 | 87.44 |
| 6′-Hydroxy justicidin A | 97.27 | 26.39 | 86.96 |
| Diphyllin apioside 5-acetate | 94.72 | 20.89 | 76.83 |
| Diphyllin apioside | 92.61 | 20.02 | 73.31 |
| Tuberculatin | 92.61 | 20.02 | 73.31 |
| Luteolin 7-glucoside | 25.17 | 4.87 | 73.28 |

28 candidate active compounds for Network pharmacology. (Note: Pharmacokinetic data below are referenced from the article by Xie et al.[15])

| **Gene Official Symbol** | | | | | |
| --- | --- | --- | --- | --- | --- |
| TNF | EGFR | MMP9 | NR3C1 | BTK | RBP4 |
| PIK3CD | STAT3 | CD38 | MMP1 | PPARG | TGFB1 |
| PDGFRB | PLA2G7 | AXL | EDNRA | NOS3 | CD81 |
| ABL1 | ELANE | F2 | MME | SERPINE1 | AGTR1 |
| KIT | ABCB1 | PLG | HRAS | IL2 | JAK2 |
| CCND1 | PTGS2 | MPO | TLR9 | PRKCD | REN |
| NOS2 | MMP2 | PIK3R1 | SELL | TP53 | CXCR3 |
| SYK | MAPK1 | AKT1 | SELE | VDR | CCR1 |
| P2RX7 | TERT | ZAP70 | SELP | G6PD | ITGB2 |
| FLT1 | BCL2 | ICAM1 | ITGA4 | PTPRC | CCR5 |
| JAK3 | TTR | VCAM1 | JAK1 | ITGAL | IL6 |
| ALOX5 | CFTR |  |  |  |  |

**Table S2**

68 potential targets for juechuang against chronic glomerulonephriti

|  | **Forward** | **Reverse** |
| --- | --- | --- |
| Il6 | CTTCCCTACTTCACAAGTCC | TTCCAAGATCTCCCTGAGAA |
| Ccl2 | CTACTCATTCACTGGCAAGA | TCTTGAGCTTGGTGACAAAT |
| Cxcl1 | GGCAGGGATTCACTTCAAGA | ATCTTGAGCTCGGCAGTGTT |
| Lcn2 | AACGTCACTTCCATCCTCGTC | AATCGCTCCTTCAGTTCATCG |
| Ccl7 | ACCGAGTCTGCCAACTTTCAC | TGAAAGCAGCAGCTGTGAGC |
| GAPDH | AAAGGGTCATCATCTCCGCC | AGTGATGGCATGGACTGTGG |

**Table S3**

Primer information.
